# Does Music Relieve Pain Through Distraction?

**DOI:** 10.1002/ejp.70409

**Published:** 2026-10-05

**Authors:** Yang Wang, Jeba Rezwana, Giancarlo Colloca

**Affiliations:** ^1^ Department of Pain and Translational Symptom Science University of Maryland School of Nursing Baltimore Maryland USA; ^2^ Department of Computer and Information Sciences Towson University Towson Maryland USA

This journal recently published a paper by Desbarats and colleagues entitled ‘The Hypoalgesic Effects of Music Are Independent From Distraction’. (Desbarats et al. 2026) The study examined whether music reduces pain by directing attention away from it or through other processes, such as pleasant emotions. Although distraction has long been proposed as an explanation for music‐induced analgesia, it remains unclear whether music's emotional effects contribute independently of attention. Understanding this question could help explain if music remains beneficial while people perform other activities that require attention.

Desbarats and colleagues used a within‐subjects factorial design in 59 healthy participants receiving painful heat stimulation on the forearm. Participants listened to music, scrambled music, or silence while completing visual tasks with low or high cognitive demand. The easy task required identifying an arrow's direction. The difficult task required identifying whether a letter matched one presented two letters earlier (i.e., a 2‐back working‐memory task). The hypothesis is that if music and the difficult task reduce pain through shared, limited attentional resources, they should compete. Music should therefore provide less pain relief during the difficult task.

Findings from this paper indicated that the difficult task reduced both pain intensity and unpleasantness relative to the easy task. Music reduced both outcomes relative to scrambled music, but only unpleasantness relative to silence. The interaction between auditory condition and task difficulty was not significant. Importantly, Bayesian comparisons further favoured an additive model, in which the effect of auditory condition did not vary with task difficulty. This pattern was interpreted as supporting an emotional contribution independent of distraction.

A strength of this study is that the study manipulated cognitive demand during music listening, directly testing whether task difficulty changes music's effects on pain. Comparing all conditions within the same participants helps account for individual differences in pain sensitivity and responses to music. Measuring both intensity and unpleasantness also helps distinguish whether music changes how strong the pain feels, how distressing it is, or both. These features make the study informative for understanding music‐induced analgesia.

However, additive effects on pain ratings do not necessarily support separate mechanisms. 2‐back performance confirms that the manipulation increased cognitive demand, but the task may not exhaust or sufficiently compete for the attentional resources through which music might influence pain. In other words, participants might still have enough attention available for music to provide further distraction. The visual task may also interfere only partly with attention to sounds.

Music and cognitive distraction may also begin with different psychological processes while influencing pain through partly shared downstream pathways. A demanding working‐memory task has been shown to reduce pain‐related spinal cord activity, with endogenous opioid mechanisms contributing to this effect. (Sprenger et al. 2012) Music listening has also been associated with changes in brainstem and spinal cord activity consistent with descending pain modulation. (Dobek et al. 2014) Thus, music and cognitive tasks may differ in their initial processes, such as emotion and attention, while converging on overlapping pain‐modulatory systems. These findings do not indicate that the mechanisms are identical, but they further suggest that additive effects on pain ratings should not be interpreted as direct evidence that the underlying mechanisms are independent.

Attention and emotion may also influence each other. Enjoyable music could draw attention away from pain, while paying closer attention to music could increase enjoyment. Feeling calmer might make pain less threatening and easier to ignore. Thus, an emotional contribution to pain relief may itself involve changes in attention.

Overall, the study provides good evidence that music‐induced analgesia was not attenuated by the particular increase in cognitive demand. Further studies remain valuable for determining when music provides additional relief and whether active listening is necessary. Including a no‐task condition in addition to easy and demanding cognitive‐task conditions would help separate the analgesic effects of music itself from distraction. Moreover, studies should also manipulate attention to music while measuring positive affect and expectations of pain relief. Clinically, this work could help determine whether patients should listen before a procedure, focus on music during it, or use it in the background. Assessing pain relief alongside the ability to follow instructions or participate in treatment would help identify how music can be used effectively in clinical care.

## Author Contributions

This commentary was developed by Y.W. and critically examined by J.R. and G.C. All authors have approved the final version and agree to be accountable for all aspects of the work.

## Linked Articles

This article is linked to Desbarats et al. papers. To view this article, visit https://doi.org/10.1002/ejp.70372.
